# Non-alcoholic and alcoholic Fatty Liver Disease - two Diseases of Affluence associated with the Metabolic Syndrome and Type 2 Diabetes: the FIN-D2D Survey

**DOI:** 10.1186/1471-2458-10-237

**Published:** 2010-05-10

**Authors:** Anna Kotronen, Hannele Yki-Järvinen, Satu Männistö, Liisa Saarikoski, Eeva Korpi-Hyövälti, Heikki Oksa, Juha Saltevo, Timo Saaristo, Jouko Sundvall, Jaakko Tuomilehto, Markku Peltonen

**Affiliations:** 1 Diabetes Prevention Unit, National Institute for Health and Welfare, Helsinki, Finland; 2 Department of Medicine, Division of Diabetes, University of Helsinki, Helsinki, Finland; 3 Minerva Medical Research Institute, Helsinki, Finland; 4 Department of Chronic Disease Prevention, National Institute for Health and Welfare, Helsinki, Finland; 5 Department of Internal Medicine, South Ostrobothnia Central Hospital, Seinäjoki, Finland; 6 Tampere University Hospital, Tampere, Finland; 7 Department of Internal Medicine, Central Finland Central Hospital, Jyväskylä, Finland; 8 Laboratory of Analytical Biochemistry, Department of Health and Functional Capacity, National Institute for Health and Welfare, Helsinki Finland; 9 Department of Public Health, University of Helsinki, Finland

## Abstract

**Background:**

Non-alcoholic fatty liver disease (NAFLD) is known to be associated with the metabolic syndrome (MetS) and abnormal glucose tolerance. Whether alcoholic fatty liver disease (AFLD) is associated with similar metabolic abnormalities has not been examined in a population-based study. We aimed at assessing the prevalences of NAFLD and AFLD, and to examine to what extent these conditions are associated with MetS and abnormal glucose tolerance.

**Methods:**

The cohort included 2766 Finnish subjects (45-74 years) from the population-based FIN-D2D survey. Features of insulin resistance, components of the MetS, glucose tolerance status by oral glucose tolerance test, serum liver enzyme concentrations, and daily alcohol consumption were assessed.

**Results:**

Subjects with NAFLD and AFLD were equally obese and had similar fasting and insulin concentrations. The prevalences of NAFLD and AFLD were 21% (95% CI: 19%-22%) and 7% (95% CI: 6%-8%). The MetS was slightly more prevalent in AFLD (73%) than in NAFLD (70%, p = 0.028), and type 2 diabetes was similarly prevalent in NAFLD and AFLD (24-25%). The MetS and type 2 diabetes were more prevalent in subjects with NAFLD or AFLD compared to subjects with normal LFTs (53% and 14%, p < 0.0001 for both).

**Discussion and conclusion:**

In Finnish middle-aged population, the prevalence of NAFLD is 3-fold higher than that of AFLD. The prevalences of MetS and type 2 diabetes are, however, significantly increased in both NAFLD and AFLD compared to subjects with normal LFTs. Subjects with AFLD are thus similarly metabolically unhealthy as subjects with NAFLD.

## Background

Non-alcoholic fatty liver disease (NAFLD) is defined as a fatty liver (liver fat >5-10% of liver weight), which is not due to excess alcohol consumption or other causes of steatosis [1]. NAFLD is associated with obesity, the metabolic syndrome, dyslipidemia, insulin resistance, and type 2 diabetes [1]. NAFLD is the most common cause of elevated liver function tests (LFTs) in the US according to the NHANES III survey [2]. Population-based studies from the US have reported the prevalence of increased liver fat content to be 34% when measured using quantitative proton magnetic resonance spectroscopy [3] and ~20% when estimated using elevated LFTs [4,5]. However, excess alcohol consumption is common and could coexist with NAFLD. Merely focusing on NAFLD by excluding subjects using excessive amounts of alcohol may thus underestimate the prevalence of the MetS and type 2 diabetes in subjects with elevated LFTs. This would seem particularly important as the long-term prognosis both with respect to the development of cirrhosis and total mortality is much worse for patients with AFLD than with NAFLD [6,7]. There are, however, no population-based epidemiological studies investigating the prevalences of both non-alcoholic and alcoholic fatty liver diseases (AFLD), and the metabolic abnormalities associated with these conditions [6].

In the clinic, it is difficult to distinguish between subjects with NAFLD and AFLD using abnormal liver function tests (LFTs). Increased serum γGT concentration is a marker of excessive alcohol consumption [8], but serum γGT concentrations are also increased in NAFLD [9]. There are no population-based data on how serum ALT, AST and γGT concentrations, or the AST/ALT-ratio may help in distinguishing between NAFLD and AFLD.

In the present study, we examined the prevalence and metabolic abnormalities of NAFLD and AFLD in a large population-based cohort of 2766 individuals in which features of insulin resistance, glucose tolerance status, and serum liver enzyme concentrations were measured. Information on alcohol consumption was assessed using self-administered questionnaire.

## Methods

### Subjects

The FIN-D2D survey was carried out in the hospital districts of Pirkanmaa, the Southern Ostrobothnia, and Central Finland between October and December 2007. A random sample of 4500 subjects aged 45-74 years, stratified according to gender, 10-year age groups (45-54, 55-64, and 65-74 years), and the three geographical areas, was selected from the National Population Register in August 2007. The study participants were invited to a clinical examination by mail. The overall participation rate was 64%. Subjects for which fasting blood samples or reliable 2-hour post-challenge glucose results in the OGTT were unavailable were excluded from the analyses (n = 39). In addition, subjects without data on alcohol consumption were excluded (n = 63). The total number of individuals included was thus 2766 (61% of the original sample).

The study protocol was approved by the Ethical Committee of the Hospital District of Helsinki and Uusimaa. All participants gave their written informed consent prior to participation in the study.

### Design

The health examination was carried out according to the WHO MONICA project [10] and the WHO Expert Group for glucose assessments. After an overnight fast, blood samples were drawn for measurement of fasting plasma (fP) glucose, fasting serum (fS) insulin, fS-triglycerides, fS-HDL and LDL cholesterol, fS-ALT, fS-AST, and fS-γGT concentrations. Height, weight, waist circumference, and body fat % were measured by nurses specially trained for the survey procedures. Body composition was measured using the Tanita TBF-300MA body composition analyzer (Tanita Corporation Tokyo Japan) based on the principle of single frequency leg-to-leg bioelectrical impedance.

An oral glucose tolerance test (OGTT) was performed in all subjects except for those with previously diagnosed diabetes (either type 1 or type 2). Alcohol consumption during the past week and the past year was assessed with a self-administered questionnaire. The average daily alcohol consumption (g/d) was calculated from the number of drinks during the past week. The results remained essentially unchanged if the average alcohol consumption was calculated from the number of drinks during the past year. The participants were asked whether they were using lipid lowering, antihypertensive, or antihyperglycemic medications. According to the Finnish Red Cross, the prevalences of hepatitis B and C were 0.03% and 0.05% among blood donors (n = 208305) during 1998-2007 including these regions of Finland (Tom Krusius, the Finnish Red Cross).

### Definitions

The cutoffs of fS-ALT or fS-AST concentrations were determined using data of 271 non-diabetic Finnish subjects [11], in which liver fat content has been directly measured using proton magnetic resonance spectroscopy. The regression coefficients and their 95% confidence intervals of relationships between liver fat % and S-ALT were 0.43 (0.29-0.55) and 0.46 (0.29-0.60) in women and men, respectively. The regression coefficients and their 95% confidence intervals of relationships between liver fat % and S-AST were 0.25 (0.09-0.39) and 0.31 (0.12-0.48) in women and men, respectively. Increased liver fat content was defined as liver fat >5.6% (based on the Dallas Heart Study [3]) and corresponded to AST concentrations of 33 U/l and 29 U/l in men and women, and to ALT concentrations of 43 U/l and 30 U/l, respectively. Men and women with increased fS-ALT or/and fS-AST concentrations consuming ≤ 20 g (for men) and ≤ 10 g (for women) of ethanol per day were considered to have NAFLD [1], while those consuming >20 g (for men) and >10 g (for women) of ethanol per day were considered to have AFLD. Additionally, the analyses were validated using higher cut-off values (30 g and 20 g of ethanol per day in men and women, respectively) for use of alcohol.

Glucose tolerance was classified according to the WHO 1999 criteria [12]. The OGTT was performed according to the WHO recommendations [12]. Fasting and 2-hour samples for measurement of plasma glucose concentrations were drawn into fluoride citrate tubes and centrifuged within 30 minutes.

The metabolic syndrome was defined according to criteria of the International Diabetes Federation [13]: central obesity (waist circumference ≥94 cm in men and ≥80 cm in women) and at least two of the following factors: 1) serum triglycerides ≥1.70 mmol/l or specific treatment for this lipid abnormality; 2) serum HDL cholesterol <1.03 mmol/l in men and <1.29 mmol/l in women or specific treatment for this lipid abnormality; 3) systolic BP ≥130 mmHg or diastolic BP ≥85 mmHg or treatment for previously diagnosed hypertension; 4) fasting plasma glucose ≥5.6 mmol/l or previously diagnosed type 2 diabetes.

### Analytical procedures and other measurements

All assays were performed at the Laboratory of Analytical Biochemistry at the National Public Health Institute, Helsinki, using Architect ci8200 analyzer (Abbott Laboratories, Abbott Park, IL). Plasma glucose was determined with a hexokinase method (Abbott Laboratories, Abbott Park, IL) and serum insulin with a chemiluminescent microparticle immunoassay (Abbott Laboratories, Abbott Park, IL). Serum total and HDL cholesterol, and triglyceride concentrations were measured with enzymatic kits from Abbott Laboratories (Abbott Park, IL). The concentrations of LDL cholesterol were calculated using the Friedewald formula [14]. Serum ALT, AST, and γGT concentrations were determined using photometric IFCC (International Federation of Clinical Chemistry) methods (Abbott Laboratories, Abbott Park, IL).

Height was measured to the nearest 0.1 cm. Weight was measured in light clothing. BMI was calculated as weight (kg) divided by height^2 ^(m^2^). Blood pressure was measured in a sitting position after a minimum of 15 minutes of acclimatization and before blood sampling using a mercury sphygmomanometer.

### Statistical analyses

Characteristics of the study population are given as means and standard deviations (SD). The differences in clinical and laboratory data between the groups in Additional file 1 were compared using analysis of covariance for continuous variables, and logistic regression analysis for dichotomous variables. Prevalences of elevated LFTs were compared with logistic regression models, using likelihood-ratio tests. These analyses were additionally adjusted for age, gender, and BMI. Analyses were performed with the statistics package Stata version 9.2 [15].

## Results

### Prevalence of elevated liver function tests (LFTs)

**Figure 1:**
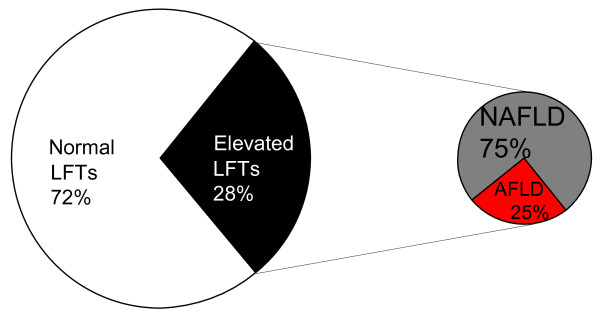
**The prevalence of elevated LFTs due to non-alcoholic (NAFLD) and alcoholic (AFLD) causes in 2766 Finnish subjects**.

Elevated LFTs were present in 28% (95% CI: 26%-29%) of the subjects. The prevalences of NAFLD and AFLD were 21% (95% CI: 19%-22%) and 7% (95% CI: 6%-8%) (Figure 1). Characteristics of subjects with and without elevated LFTs and either low or high alcohol consumption are shown in Additional file 1. Clinical and biochemical characteristics of subjects with normal LFTs were essentially similar in those with low or high alcohol consumption. Subjects with NAFLD had similar BMI, waist, whole body fat %, fP-glucose, 2h-glucose, and fS-insulin concentrations as subjects with AFLD (Additional file 1). The AST/ALT-ratio was similar, fS-ALT 9%, fS-AST 12%, and S-γGT concentrations 43% higher in AFLD as compared to NAFLD (Additional file 1, Figure 2).

**Figure 2:**
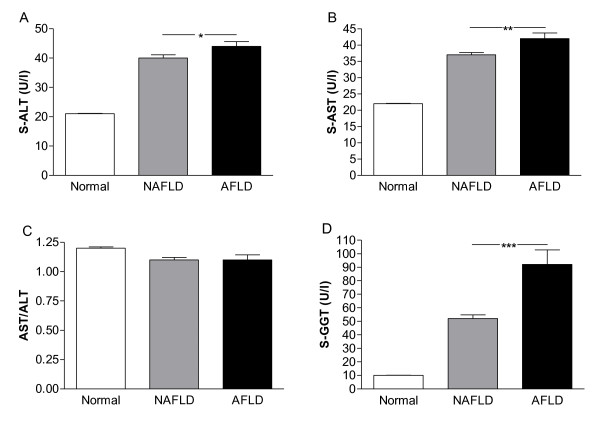
**(A) S-ALT concentrations, (B) S-AST concentrations, (C) AST/ALT-ratio, and (D) S-γGT concentrations in subjects with normal LFTs [subjects with low (n = 1691) or high (n = 308) alcohol consumption, Additional file **1**], NAFLD, and AFLD**. For differences between normal LFTs vs. NAFLD or AFLD, please see Additional file 1. *p < 0.05, *p < 0.01, ***p < 0.001. The error-bars are standard error of the mean.

### NAFLD and AFLD in the metabolic syndrome, IGT, and type 2 diabetes

**Figure 3:**
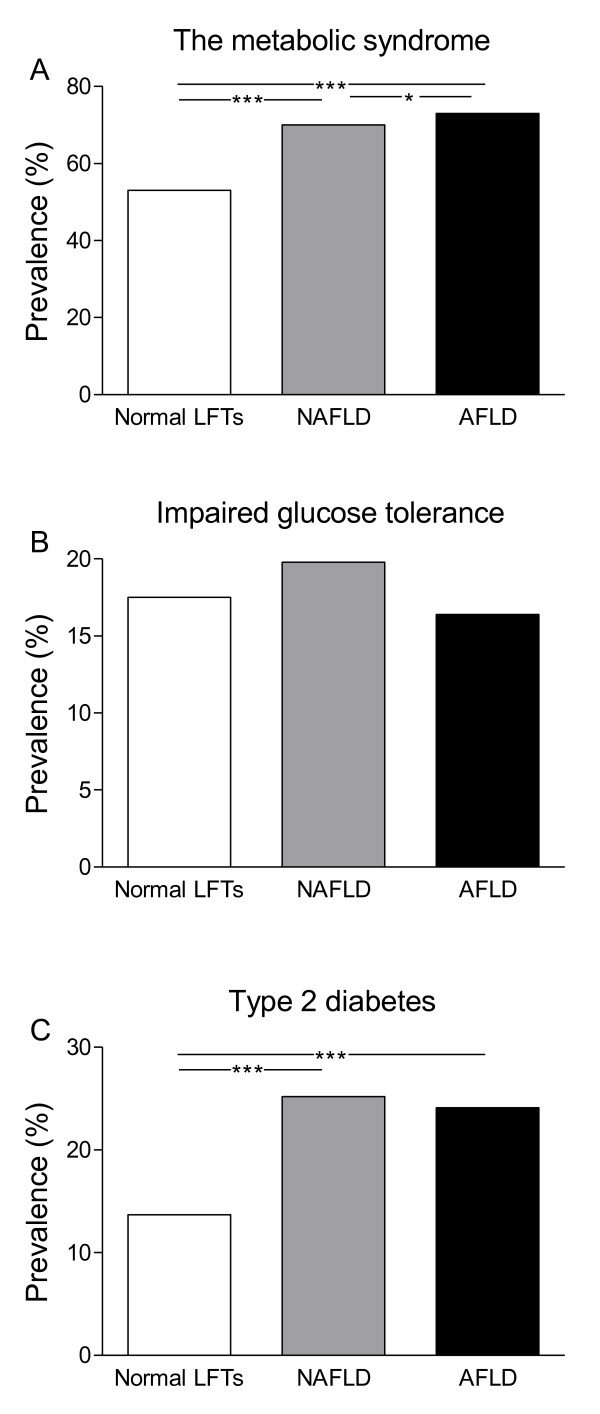
**The prevalences of (A) the metabolic syndrome, (B) impaired glucose tolerance, and (C) type 2 diabetes in subjects with normal LFTs [subjects with low (n = 1691) or high (n = 308) alcohol consumption, Additional file **1**], NAFLD, and AFLD**. *p < 0.05, **p < 0.01, ***p < 0.001.

In subjects with NAFLD and AFLD, the prevalences of the metabolic syndrome were 70% and 73% (p = 0.028, NAFLD vs. AFLD, Figure 3a). These prevalences were significantly higher than in subjects with normal LFTs (53% in subjects with low and high alcohol consumption, p = 0.001 for both, p = 0.01 after adjusting for age, gender, and BMI). The prevalence of IGT was 20% in NAFLD and 16% in AFLD (NS, NAFLD vs. AFLD, Figure 3b), which was not different from subjects with normal LFTs (18%). Type 2 diabetes was present in 25% of subjects with NAFLD and 24% of subjects with AFLD (NS, NAFLD vs. AFLD, Figure 3c). These prevalences were significantly higher compared to subjects with normal LFTs (14%, p < 0.001 for both, p < 0.0001 after adjusting for age, gender, and BMI).

### The metabolic syndrome, IGT, and type 2 diabetes in NAFLD and AFLD

**Figure 4:**
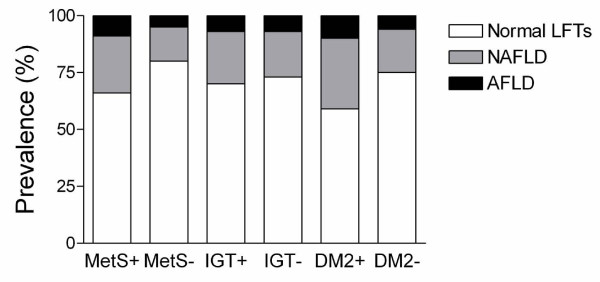
**The prevalences of NAFLD and AFLD in subjects with (MetS+) and without (MetS-) the metabolic syndrome, impaired glucose tolerance (IGT), and type 2 diabetes (T2D)**. Please see text for significances.

In subjects with the metabolic syndrome, the prevalences of NAFLD and AFLD were significantly higher as compared to those without the syndrome (p = 0.001 for both, p = 0.02 after adjusting for age, gender, and BMI, Figure 4). The prevalences of NAFLD and AFLD were similar in subjects with and without IGT. In subjects with type 2 diabetes, the prevalences of NAFLD and AFLD were significantly higher as compared to subjects without type 2 diabetes (p = 0.01 for both, p = 0.01 after adjusting for age, gender, and BMI).

When the above-described analyses were replicated by defining NAFLD and ALFD using higher cut-off values for alcohol use (30 g and 20 g of ethanol per day in men and women, respectively), the main results comparing subjects with normal LFTs, NALFD and AFLD, remained essentially unchanged.

## Discussion

To our knowledge, this is the first population-based study comparing prevalences and associated metabolic abnormalities between NAFLD and AFLD. In the present study, the prevalence of NAFLD was 21% when estimated using elevated LFTs, in keeping with recent population-based surveys in which the prevalence of NAFLD was estimated using abnormal LFTs [5]. The metabolic syndrome and type 2 diabetes in AFLD were as prevalent as in NAFLD.

In NAFLD, LFTs are only mildly or moderately elevated. We therefore did not use cut-offs of elevated liver enzymes from routine laboratory (S-ALT > 70 U/l in men and > 45 U/l in women, S-AST > 45 U/l in men and > 35 U/l in women) but rather cut-off values which in our laboratory corresponded to increased liver fat content measured with ^1^H-MRS (>5.6% [3]). As these cut-off values are based on estimated associations between liver fat and liver enzymes in a study sample, there is some inheritent uncertainty in the diagnostic limits, as illustrated by the confidence intervals for the estimates of regression coefficients. However, these cut-off values were almost identical to those suggested by Prati et al [16] for identification of subjects with liver disease. Both the AST/ALT-ratio [8] and serum γGT concentrations [8,17] have been suggested to be more specific to alcoholic liver disease than NAFLD [18-21]. In the present study, subjects with AFLD consumed 10-times more alcohol than subjects with NAFLD, but there were no clinically significant differences in S-ALT, S-AST, or AST/ALT-ratio between the two groups. S-γGT concentrations were ~2-fold higher in AFLD compared to NAFLD, confirming that S-γGT is a useful marker of alcohol-induced liver disease.

Approximately one third of the population worldwide has the metabolic syndrome [22]. The prevalence increases with age [22]. In our cohort of 45-74 yrs old subjects, approximately 70% of subjects with NAFLD had the metabolic syndrome, which was significantly higher than in subjects with normal LFTs (53%). Due to a large sample size, there is a potential to identify very small differences between the groups. Some of the differences may not be of a clinical significance, but differences in BMI, liver enzymes, triglyceride and fasting glucose concentrations between subjects with and without fatty liver disease should be considered as clinically relevant. In the present study, the AFLD and NAFLD groups were comparable with respect to BMI, waist circumference, % body fat and glucose and insulin concentrations (Additional file 1). Despite nearly similar prevalences of the metabolic syndrome, the AFLD group had significantly higher HDL cholesterol and higher triglycerides than the NAFLD group (Additional file 1). In the face of otherwise matched groups, these lipid differences are likely to be due to alcohol, which is known to increase both HDL cholesterol and triglyceride concentrations [23,24]. Diastolic blood pressure was also significantly higher in AFLD than in NAFLD, which could also be due to alcohol use independent of obesity and insulin [25].

NAFLD predicts type 2 diabetes independent of obesity [26]. A small study of 114 subjects with bright liver and chronically elevated LFTs suggested that approximately half of subjects with NAFLD have impaired or diabetic glucose tolerance [27]. We found the prevalence of type 2 diabetes to be 25% in NAFLD and 24% in AFLD, which was significantly higher than in subjects with normal LFTs (14%). In previous studies of subjects with biopsy-proven NAFLD, the prevalence of type 2 diabetes has ranged from 20 to 50% [5,28]. These data suggest that an OGTT should be considered in subjects with even mildly elevated LFTs.

The strengths of the present study include a population-based approach and a large and representative sample of middle-aged individuals studied in three districts of Finland where the prevalence of hepatitis is low. In addition, all measurements were performed centrally using the same methods. However, our study has several limitations. First, self-reported alcohol consumption is frequently subject to underreporting. We therefore used low limits of alcohol consumption to separate subjects with NAFLD and AFLD [1]. In addition, the results were essentially similar if alcohol consumption was calculated from the number of drinks during the past week or year. Second, although we used low cut-offs of ALT and/or AST to diagnose NAFLD, it is likely that not all subjects with NAFLD or AFLD were identified due to poor sensitivity of LFTs. This is an epidemiological study on 2766 subjects and measuring liver fat content with proton magnetic resonance spectroscopy is a challenge in such a large study population. Third, the cut-off of ethanol intake is valid to define NAFLD, but values above the cut-off do not necessarily indicate AFLD. Whenever drinking is limited to social drinking and is accompanied to excess energy intake, it is largely possible that in most patients with alcohol intake above the safe limits, AFLD and a metabolic liver disease unrelated to alcohol may coexist. Forth, the participation rate of subjects who consume excess alcohol might be low, which might underestimate the true prevalence of AFLD.

## Conclusion

In conclusion, when studied in a population-based survey, AFLD is less prevalent than NAFLD, but the metabolic syndrome and type 2 diabetes are equally common in these conditions. Thus, it is important to recognize and treat the metabolic abnormalities not only in subjects with low but also in those with high alcohol consumption and mildly elevated LFTs.

## Abbreviations

AFLD: alcoholic fatty liver disease; ALT: alanine aminotransferase; AROC: area under the ROC curve; AST: aspartate aminotransferase; BP: blood pressure; BMI: body mass index; fP: fasting plasma; fS: fasting serum; γGT: gamma glutamyltransferase; HbA_1c_: glycosylated hemoglobin 1c; HDL: high density lipoprotein; ^1^H-MRS: proton magnetic resonance spectroscopy; IGT: impaired glucose tolerance; LDL: low-density lipoprotein; LFT: liver function test; MetS: metabolic syndrome; NAFL: non-alcoholic fatty liver; NAFLD: non-alcoholic fatty liver disease; NASH: non-alcoholic steatohepatitis; NHANES: Third National Health and Nutrition Examination Survey; OGTT: oral glucose tolerance test; T2D: type 2 diabetes.

## Competing interests

The authors declare that they have no competing interests.

## Authors' contributions

AK, HY-J, SM, EK-H, HO, J Saltevo, TS, JT, and MP participated in the design of the study; MP performed the statistical analysis; LS and J Sundvall participated in acquisition of the data; AK, HY-J, and MP participated in the interpretation of the data and drafted the manuscript. All authors read and approved the final manuscript.

## Pre-publication history

The pre-publication history for this paper can be accessed here:

http://www.biomedcentral.com/1471-2458/10/237/prepub

## Supplementary Material

Additional file 1 Table: Characteristics of subjects with and without NAFLD or AFLD. Click here for file
