# Supplementary material for: Non-alcoholic and alcoholic Fatty Liver Disease - two Diseases of Affluence associated with the Metabolic Syndrome and Type 2 Diabetes: the FIN-D2D Survey
Source: BMC Public Health. 2010 May 10;10:237. doi: 10.1186/1471-2458-10-237 (PMC2873937; doi:10.1186/1471-2458-10-237)
Supplement: Additional file 1 — Table: Characteristics of subjects with and without NAFLD or AFLD. [file 1471-2458-10-237-S1.DOC]

**Table 1.** Characteristics of subjects with and without NAFLD or AFLD.

|  | **Low alcohol consumption** | |  | **High alcohol consumption** | |  | **Comparison of NAFLD vs. AFLD** | |
| --- | --- | --- | --- | --- | --- | --- | --- | --- |
|  | **Normal LFTs** | **NAFLD** | **p-value** | **Normal LFTs** | **AFLD** | **p-value** | **p-value** | **p-value adjusted for gender:** |
| n (% women) | 1691 (51%) | 572 (60%) | <0.001 | 308 (52%) | 195 (42%) | 0.037 | <0.001 | - |
| Age (yrs) | 61±9 | 60±8 | NS | 56±8 | 57±7 | NS | <0.001 | <0.001 |
| **Body composition** |  |  |  |  |  |  |  |  |
| BMI (kg/m^2^) | 27.0±4.3 | 29.2±5.7 | <0.001 | 26.6±4.6 | 28.8±5.1 | <0.001 | NS | NS |
| Waist (cm) | 94±13 | 95±15 | <0.001 | 93±14 | 101±14 | <0.001 | 0.012 | NS |
| Whole body fat % | 31.5±8.5 | 35.4±8.8 | <0.001 | 31.2±8.5 | 33.4±8.7 | 0.007 | 0.005 | NS |
| **P-glucose and fS-insulin** |  |  |  |  |  |  |  |  |
| fP-glucose (mmol/l) | 6.1±1.1 | 6.5±1.4 | <0.001 | 6.1±0.8 | 6.5±1.4 | <0.001 | NS | NS |
| 2h glucose (mmol/l) | 6.9±2.6 | 7.8±3.1 | <0.001 | 6.4±2.2 | 7.6±3.0 | <0.001 | NS | NS |
| fS-insulin (mU/l) | 8±19 | 11±15 | <0.001 | 7±4 | 10±8 | <0.001 | NS | NS |
| **Blood pressure** |  |  |  |  |  |  |  |  |
| Systolic BP | 137±19 | 139±18 | 0.016 | 132±17 | 140±18 | <0.001 | NS | NS |
| Diastolic BP | 81±10 | 82±9 | NS | 81±9 | 85±9 | <0.001 | <0.001 | <0.001 |
| **Serum lipids** |  |  |  |  |  |  |  |  |
| fS-triglycerides (mmol/l) | 1.29±0.63 | 1.51±0.75 | <0.001 | 1.28±0.67 | 1.89±1.86 | <0.001 | 0.005 | 0.011 |
| fS-HDL cholesterol (mmol/l) | 1.43±0.33 | 1.40±0.37 | NS | 1.56±0.33 | 1.50±0.41 | NS | 0.003 | <0.001 |
| fS-LDL cholesterol (mmol/l) | 3.39±0.85 | 3.35±0.92 | NS | 3.34±0.80 | 3.43±0.83 | NS | NS | NS |
| **Liver enzymes** |  |  |  |  |  |  |  |  |
| S-ALT (U/l) | 21±7 | 40±27 | <0.001 | 21±7 | 44±23 | <0.001 | 0.042 | NS |
| S-AST (U/l) | 22±5 | 37±17 | <0.001 | 22±5 | 42±24 | <0.001 | 0.002 | 0.012 |
| AST/ALT | 1.2±0.5 | 1.1±0.5 | 0.004 | 1.1±0.5 | 1.1±0.6 | NS | NS | NS |
| S-γGT (U/l) | 27±18 | 52±63 | <0.001 | 37±28 | 92±149 | <0.001 | <0.001 | 0.001 |
| Alcohol consumption  (g/d) | 4±5 | 4±5 | NS | 26±17 | 32±21 | 0.001 | <0.001 | <0.001 |
| Self-reported use of lipid-lowering drugs (%) | 23.5 | 30.2 | 0.002 | 15.0 | 21.9 | NS | 0.035 | 0.023 |
| Self-reported use of antihypertensive drugs (%) | 31.3 | 41.1 | <0.001 | 24.9 | 38.0 | 0.002 | NS | NS |

Data are shown as mean ± SD.
